# Apple Fibers as Carriers of Blackberry Juice Polyphenols: Development of Natural Functional Food Additives

**DOI:** 10.3390/molecules27093029

**Published:** 2022-05-08

**Authors:** Ivana Buljeta, Mario Nosić, Anita Pichler, Ivana Ivić, Josip Šimunović, Mirela Kopjar

**Affiliations:** 1 Faculty of Food Technology Osijek, Josip Juraj Strossmayer University of Osijek, F. Kuhača 18, 31000 Osijek, Croatia; ivana.buljeta@ptfos.hr (I.B.); m.nosic@yahoo.com (M.N.); anita.pichler@ptfos.hr (A.P.); ivana.ivic@ptfos.hr (I.I.); 2 Department of Food, Bioprocessing and Nutrition Sciences, North Carolina State University, Raleigh, NC 27695, USA; simun@ncsu.edu

**Keywords:** anthocyanins, apple fibers, freeze-drying, antioxidants, food additives

## Abstract

Blackberry polyphenols possess various health-promoting properties. Since they are very sensitive to environmental conditions such as the presence of light, oxygen and high temperatures, the application of such compounds is restricted. Fibers are recognized as efficient carriers of polyphenols and are often used in polyphenols encapsulation. In the present study, the ability of apple fiber to adsorb blackberry juice polyphenols was examined. Freeze-dried apple fiber/blackberry juice complexes were prepared with different amounts of fibers (1%, 2%, 4%, 6%, 8% and 10%) and a constant amount of blackberry juice. Polyphenol profile, antioxidant activity, inhibition of the α-amylase, color parameters, as well as the IR spectra, of the obtained complexes were assessed. The results showed a negative effect of higher amounts of fiber (more than 2%) on the adsorption of polyphenols and the antioxidant activity of complexes. With the proper formulation, apple fibers can serve as polyphenol carriers, and thus the application as novel food additives can be considered.

## 1. Introduction

Dietary fibers are well known for their health-promoting potentials such as lowering blood cholesterol and sugar levels and decreasing the risk of development of coronary diseases, hypertension, diabetes, obesity and gastrointestinal disorders [1,2]. In addition, they have high potential as food additives or as functional food ingredients. They can meet the technological and functional purposes necessary for the development of health-promoting value-added products [1,2]. Functional fibers can be further used as thickeners, oil carriers, texturizers and moisture retention agents in yogurts, ice creams, sauces, dressings, beverages, meat products and baked goods [3,4]. Apples are considered the most consumed fruit (around 13% of all fruits consumed) [5,6,7]. In addition to their consumption in the fresh form, they are used as raw materials for food products whose production leads to considerable amounts of by-products. The potential applications of these by-products have been traditionally underestimated. Currently, by-products of apple processing are receiving increasing attention as a rich source of bioactive compounds [5]. As a functional ingredient and source of fibers, they are used in the production of bakery products such as breads, cakes and biscuits.

Changes in lifestyle, eating habits and modifications in the nutritional demands have led to important changes in the food industry in recent decades. Additional food ingredients that are incorporated into the food products are widely used and can improve sensory, microbiological and physicochemical properties, increase shelf-life and are beneficial for human health [6]. As the consumers’ awareness of the connection between food and health is on the rise, the focus is on the natural compounds and their associated bioactivities as food ingredients and/or food additives [8]. Due to these reasons, for the present formulation of functional food ingredients, apple fibers, containing cellulose, hemicellulose and pectin, were chosen. Blackberry juice was selected as a source of polyphenols since blackberries are rich in these bioactive compounds [9]. Among all anthocyanins present in blackberry juice, cyanidin 3-glucoside is dominant (around 92% of all anthocyanins in blackberries) and contributes to the dark blue color of this fruit/juice. There are many health benefits associated with anthocyanins intake such as reduction in insulin resistance, anti-inflammatory, anti-obesity effects, prevention of cardiovascular diseases, etc. Among phenolic acids, ellagic acid is primary in blackberries (the highest concentrations in the seeds). Studies indicate a positive impact of ellagic acid metabolites on reduced risk of cancer [9,10]. Phytochemicals such as polyphenols have been studied due to their potential biological activity associated with their antioxidant protection and positive influence on food quality [11].

There are studies on the interactions between dietary fibers and polyphenols. Strawberry dietary fibers have been used as carriers of hydroxytyrosol and 3,4-dihydroxyphenylglycol for efficient preparation of functional food ingredients, which could potentially promote intestinal health since both polyphenols are antioxidants with important biological activity [12]. Also, these compounds were complexed with pectin in order to obtain complexes with potential use for colon targeting [13,14]. Interactions between apple cell walls and native polyphenols revealed that procyanidins bound to components of cell walls while the opposite effect was determined for hydroxycinnamic acids and epicatechin [15]. Apple cell walls and their polysaccharides affected the antioxidant activity of quercetin [16] while fiber from onions did not affect it [17]. A study on the utilization of cellulose and pectin as cell wall models showed that those compounds can bind anthocyanins and phenolic acids [18,19]. Also, it was observed that cellulose can be used as an efficient carrier of raspberry polyphenols and such obtained complexes can be used as colorants in the food industry [20].

The objective of this study was to encapsulate blackberry juice polyphenols onto apple fiber by a freeze-drying technique. It is known that freeze-drying has disadvantages such as higher unit costs. Despite that, it is widely used for obtaining high-value food products. By comparing freeze-drying with a second commonly used method for encapsulation, spray drying (which is more cost-effective than freeze-drying), many authors obtained higher retention of polyphenols by freeze-drying. Also, storage of freeze-dried encapsulates is safer and in some cases almost without degradation of polyphenols. Although a low-cost by-product (apple fibers) is used, the final product, freeze-dried powder enriched with polyphenols and volatiles, presents a highly valuable food additive [21,22]. All formulated complexes were evaluated for total polyphenols, proanthocyanidins, individual polyphenols, antioxidant activity, inhibition of α-amylase, color parameters and flavor profile. Additionally, IR spectra were recorded. The following hypotheses were investigated in the present study: (i) apple fiber can be applied for encapsulation of blackberry juice polyphenols, (ii) the amount of used fiber (1%, 2%, 4%, 6%, 8% and 10%) in complexes affects the retention of polyphenols and (iii) obtained complexes possess antioxidant activity and inhibitory capability towards α-amylase. To the best of our knowledge, this is the first study that deals with the incorporation of blackberry juice polyphenols in apple fibers. Consequently, understanding of interactions between apple fibers and blackberry juice polyphenols should contribute to the development of functional food additives. Obtained bioactive complexes could be applied in different food products, for example, baked goods, smoothies and shakes.

## 2. Results and Discussion

### 2.1. Evaluation of Polyphenols

In Table 1 the results of total polyphenols and proanthocyanidins present in complexes are shown. The results of total polyphenols ranged from 1.18 g GAE/100 g to 1.82 g GAE/100 g. The highest values was observed in complex with 1% of fiber while complexes with higher amounts of fiber possessed a lower reducing capability. It was observed that complexes with lower amounts of fiber had higher total polyphenols content. The same trend was observed for total proanthocyanidins content. These concentrations in complexes ranged from 57.43 mg/100 g to 98.85 mg/100 g. In complexes with 1% and 2% of fiber, the highest concentrations of total proanthocyanidins were observed with no statistical difference between these samples. With the increased amount of fiber, proanthocyanidin content decreased thus complex with 10% of fiber had 40% lower proanthocyanidin concentrations compared to the complex with 1% of fiber.

Interactions between proanthocyanidins with fibers from cell wall material are dependent on proanthocyanidins concentration, molecular mass, degree of galloylation and polymerization as well as cell wall composition and structure [23]. Results of some studies showed that a higher concentration of polyphenols present in the surroundings increased the adsorption of proanthocyanidins [15,23]. Regarding the proanthocyanidins properties, the ones with (+)-catechin units showed higher affinity to polysaccharides than the ones with (−)-epicatechin extension units [23]. The impact of cell wall composition on proanthocyanidins adsorption was also studied. The increased cell wall porosity led to the greater adsorption of higher molecular weight tannins in grape skin cell walls due to the facilitated penetration of molecules [24]. On the other hand, the reduced surface area of apple cell walls and consequently reduced porosity caused by harsh drying resulted in a decreased affinity of procyanidins to cell walls [25]. Also, according to the published literature, citrus pectins with higher contents of rhamnogalacturonans type 1 possess a higher affinity towards procyanidins than apple pectins [23]. In one of our previous studies [26], polyphenols were encapsulated with citrus fibers and total proanthocyanidins content was evaluated. These results indicated the same trend as in this study; that is, the higher amounts of citrus fiber led to a decrease in the concentration of procyanidins.

Concentrations of individual polyphenols present in blackberry juice and apple fibers, which were used for the preparation of complexes, are given in Table 2. Regarding blackberry juice, cyanidin 3-glucoside, cyanidin 3-dioxalylglucoside, quercetin, rutin, ellagic, chlorogenic, p-coumaric, caffeic and gallic acids were detected. These findings were in accordance with the previously published studies [9,27]. The identified and quantified polyphenols from apple fiber were quercetin, rutin, chlorogenic acid, two hydroxycinnamic acid derivates, phloretin and phlorizin. These compounds originated from apples and are in accordance with polyphenols usually present in apples [5].

Polyphenols present in complexes are listed in Table 3. It can be observed that cyanidin 3-glucoside was the most abundant anthocyanin in blackberry juice as well as in complexes where its concentrations ranged from 103.72 mg/100 g to 219.63 mg/100 g. The second anthocyanin present in blackberry juice, cyanidin 3-dioxalylglucoside, was detected in lower concentrations in juice and consequently in complexes (from 31.94 to 57.81 mg/100 g). The highest concentration of both anthocyanins was in the complex prepared with 2% of fiber (277.44 mg/100 g), while in complex with 1% of fiber, a lower concentration (262.36 mg/100 g) was observed. Other complexes had lower anthocyanins concentrations, and as the amount of fiber increased, this decrease became more pronounced. Quercetin and rutin were present in blackberry juice, as well as in apple fiber, but only quercetin was identified in complexes.

As the levels of used fiber increased, quercetin concentration in complexes also increased and ranged from 55.60 mg/100 g to 96.82 mg/100 g. Rutin was not detected on complexes probably because of the cleavage of the bond between rutin and apple fiber thus it was left in the supernatant. Also, its de-glycosylation and transformation into quercetin in acidic conditions is another possible reason [28]. From the class of phenolic acids, ellagic acid, chlorogenic acid and two hydroxycinnamic acid derivatives were present in complexes. Among acids, ellagic acid was present in the highest concentrations in complexes and ranged from 15.25 mg/100 g to 37.97 mg/100 g. The highest concentration of ellagic acid was measured in the complex with 1% fiber while increasing the fiber amounts resulted in the decrease of its concentration. The opposite trend was observed for chlorogenic acid. Its concentrations in complexes ranged from 17.42 mg/100 g to 26.07 mg/100 g, with the highest concentration measured in the complex with 10% of fiber. On the other hand, the two hydroxycinnamic acid derivatives ranged from 18.00 mg/100 g to 18.73 mg/100 g and from 16.10 mg/100 g to 16.60 mg/100 g, respectively. From the class of dihydrochalcones, phloretin and phlorizin originating from apple fiber were detected in complexes and ranged from 4.06 mg/100 g to 11.26 mg/100 g and from 15.23 mg/100 g to 41.86 mg/100 g, respectively. Their concentrations were affected by fiber amounts; higher fiber amounts resulted in their higher concentrations.

Previously, polyphenols and dietary fibers were studied separately. However, it has been proven that these two groups of components, found together in plants and foods, could interact. For that reason, they are studied simultaneously in order to obtain better insight into their behavior in our bodies, as well as for the preparation of functional food ingredients [15,29]. Their interactions could occur through non-covalent bonds, such as hydrogen bonds, hydrophobic interactions, and van der Waals forces, or simply by physical entrapment. Polyphenols’ affinity for polysaccharides is assumed to be strongly affected by the conformational flexibility and molecular weight of polyphenols, as well as the nature and conformation of polysaccharides [15].

Polyphenols’ hydrophilic hydroxyl groups attached to the hydrophobic aromatic ring give them the ability to bind to polysaccharides. That binding occurs between their hydroxyl groups and oxygen atoms of the glycosidic linkages of polysaccharides. Also, covalent bonds such as ester bonds can be formed between polysaccharides and phenolic acids [29]. Furthermore, these interactions could depend on surface properties. Pore size in the polysaccharides can limit the penetration of polyphenols with larger molecular masses [29]. On the other hand, some studies confirmed that polyphenols with higher molecular weight bind more to cellulose compared to those with lower molecular weight [30]. That observation could be an explanation for the absence of gallic, caffeic and p-coumaric acids, which were determined in blackberry juice but not in complexes. Polyphenols-pectin interactions have already been studied. In native pectin, hydrophobic pockets and hydrophilic domains enable the retention of polyphenols. In the case of anthocyanins, these interactions take place through hydrogen bonds between hydroxyl groups of anthocyanin B-ring and galacturonic acid in the homogalacturonan chain in pectin [31]. One study on anthocyanin binding to plant cell walls (pectin and cellulose) showed that only occasional sites on cellulose were available for binding. The authors proposed two mechanisms of interaction: direct interactions of anthocyanins with cellulose and/or pectin and stacking effects, that is, associating with previously bound anthocyanins [18]. Phan et al. [32] suggested electrostatic interactions between cyanidin 3-glucoside flavylium cations and anionic groups in the cellulose-pectin wall material and also that cellulose is a major factor controlling the binding of cyanidin 3-glucoside to models containing pectin. Furthermore, for all examined polyphenols (cyanidin 3-glucoside, (+/−)-catechin and ferulic acid) it was determined that cellulose was the main binding component regardless of other components present in systems. In a study by Bermúdez-Oria et al. [12], it was observed that interactions are dependent on the chemical structure of polyphenols. Complexation of hydroxytyrosol and 3,4,-dihydroxyphenylglycol with pectin was more pronounced for hydroxytyrosol due to the additional hydroxyl group in its structure and consequently, a higher ability to form hydrogen bonds. Interactions between plant cell walls (containing cellulose and pectin as main constituents) and polyphenols seem to occur spontaneously and immediately after contact with each other [32]. Costa et al. [11] studied the adsorption capacity of catechin, ferulic and caffeic acids acid onto xylan and cellulose. They observed that adsorption of polyphenols occurred in the first minute of contact while, after 10 minutes, there was a saturation of the active sites on the adsorbent.

Hydrogen bonds and hydrophobic interactions could be the driving forces for the binding of blackberry juice polyphenols onto apple fibers. Available binding sites on apple fibers were also considered to be an important factor in determining the amounts of bonded polyphenols. Since apple fibers used in our study contained polyphenols, maybe they reduced the available binding sites for blackberry juice polyphenols. Furthermore, during complexation polyphenols present in higher concentrations in the mixture were consequently present in higher concentrations in complexes, which could suggest concentration-dependent adsorption. The same was observed in the study of Phan et al. [30] and Jakobek et al. [33]. Adsorption of individual polyphenols from chokeberry, present in the higher concentrations in the extract (cyanidin 3-galactoside and cyanidin 3-arabinoside), led to the more pronounced adsorption onto barley β-glucan [33]. The authors proposed hydrogen bonds and Van der Waals forces as driving forces, where the creation of hydrogen bonds reduces the distance between polyphenols and β-glucan and enables the creation of van der Waals forces. From the present results, the increased amount of fiber in complexes (above 2%), negatively affected the binding of polyphenols and we assumed that the maximum binding capacity has been achieved below that point.

Awareness of the effect of synthetic materials on the environment caused an increase in the search for natural polymers that can be utilized as effective eco-friendly materials [34]. Apple pomace can be used as a sustainable source for the production of valuable polymers such as apple fibers, which can be further used in the food industry [35]. The results of our research showed that apple fibers can be good carriers of blackberry polyphenols and volatiles, proving apple fibers’ sustainability when used in the proper ratio with the source of targeted compounds. However, there are still unadsorbed valuable compounds from selected sources, in our case, blackberry juice, which can be further utilized in the food industry. They can be reused for adsorption onto apple fibers or other carries, or they can be utilized for the formulation of other types of encapsulates for example hydrogel beads.

### 2.2. Antioxidant Activity and Inhibition of α-Amylase

In Table 4, the results of all antioxidant activity assays are given. The values of antioxidant activity evaluated by the ABTS assay ranged from 73.74 µmol TE/100 g to 106.86 µmol TE/100 g. The highest value was observed for complex with 1% of fiber, while the subsequent addition of fiber resulted in a decrease in antioxidant activity. There was no statistical difference between complexes with 4% and 6% of fiber. For our complexes, antioxidant activity evaluated by the DPPH assay ranged from 88.90 µmol TE/100 g to 109.64 µmol TE/100 g. The same trend was also observed for the ABTS; the higher amounts of fiber in complexes resulted in lower antioxidant activity. However, statistical analysis showed that between complexes with 2% and 4% of fiber and 4% and 6% of fiber there was no statistical difference. The results of CUPRAC and FRAP assays followed the same trend as with the previous two methods and ranged from 687.97 µmol TE/100 g to 964.00 µmol TE/100 g and from 9.04 µmol TE/100 g to 13.19 µmol TE/100 g, respectively. From the results of all assays, it can be observed that complex with 1% of fiber had the highest values regardless of the assay used while increasing the amounts of fiber in complexes led to a decrease in antioxidant activity.

Antioxidant activity of fiber/polyphenols complexes was previously studied [20,26,36]. Considering the antioxidant activity, encapsulation of raspberry polyphenols with cellulose by freeze-drying was more efficient with lower amounts of cellulose [20]. Blackberry juice polyphenols encapsulated with citrus fibers showed a higher antioxidant potential when 1% of fiber was present in complexes, compared to 4% of fiber [26]. Da Rosa et al. [36] observed the higher antioxidant activity in microcapsules of blackberry polyphenols extract coated with xanthan (90.75%) and β-cyclodextrin (84.43%) than in chitosan (80.38%). Their results for antioxidant activity were in accordance with the amounts of polyphenols in microcapsules.

Inhibition (%) of α-amylase by application of apple fiber/blackberry juice complexes is presented in Table 4. From the results, it was noticed that the complex with 10% of fiber, in given conditions, possessed the lowest capability to inhibit α-amylase (22.10%). Furthermore, complexes with 1%, 6% and 8% of fiber showed around 33% of inhibition capability without a statistical difference among them. The highest inhibition capability was determined for the complex with 4% of fiber (37.53%) followed by the complex with 2% of fiber (35.90%). Figueiredo-Gonzálezet et. al. [37] determined that there is no positive correlation between the concentration of phenolic compounds and inhibition of α-amylase when they tested extracts of the Myrcia genus. Similar results were obtained in other studies and it was pointed out that interactions between phenolics and enzymes depend on the phenolic structure. Additionally, synergistic and/or antagonistic effects after interactions between phenolic compounds can be quite important in the inhibition of enzymes, as well as the interaction with nonphenolic compounds [38,39,40,41,42]. This trend cannot be correlated with total polyphenols and proanthocyanidin concentrations in complexes, but it can be assumed that the individual polyphenols and/or their ratios had an effect on the inhibition of α-amylase. α-amylase enzyme hydrolyses α-1,4 glycosidic linkages of alpha-linked polysaccharides (starch) and as a result, low molecular weight products (maltose, maltotriose and limit dextrins) are obtained. By inhibiting an α-amylase enzyme using natural inhibitors such as polyphenols, beneficial effects on starch digestion as well as regulation of blood glucose levels and reduction of hyperglycemia can be achieved. The clinical application can be for diminishing postprandial blood glucose levels in type-2 diabetic patients because amylase inhibitors delay the degradation of carbohydrates in the small intestine. Amylase inhibitors can also be used for controlling obesity. Due to the amylase inhibitor activity, reduction in the carbohydrate uptake lead to less calorie uptake [43,44].

### 2.3. Evaluation of Color Parameters

Color parameters of apple fiber/blackberry juice complexes are given in Table 5. In apple fiber/blackberry juice complexes the lightness ranged from 45.90 to 49.70. Complex with 1% of apple fiber had the lowest L* value, while complex with 10% had the highest value, that is, the higher amount of fiber led to the higher L* values. Positive a* values indicated the presence of red color in the complexes. The highest a* values belonged to the complexes with 4% and 6% of apple fiber (19.41 and 19.33, respectively), while the complex with 1% of apple fiber had the lowest a* value (18.03). By observing b* values in complexes, it could be noticed that samples had tones of yellow and these values ranged from 8.77 to 12.93. Results showed that with an increasing amount of fiber, the b* value also increased. For the °h and C* values, the same trend as for b* was observed and results ranged from 25.92 to 34.98 and from 20.01 to 22.54, respectively. Thus, the higher amounts of fiber caused the higher hue angle and color saturation values.

Anthocyanin pigments from blackberry juice were responsible for the red color of the complexes. These pigments are sensitive to various environmental factors and the food industry is constantly struggling with the preservation of these natural colorants. The application of anthocyanins as natural colorants in the food industry is limited due to low stability during processing and storage. For such pigments, freeze-drying is accepted as a suitable drying method that can promote easier handling, extend shelf life and improve processing stability [45,46].

### 2.4. Volatile Compounds of Apple Fiber/Blackberry Juice Complexes

The influence of apple fiber amounts on the adsorption of blackberry juice volatiles was also investigated. In most studies dealing with the investigation of the selection of efficient carriers of polyphenols from natural sources, volatile compounds are neglected. Natural sources of polyphenols such as blackberry juice also contain specific volatile compounds that are responsible for its flavor. During the complexation of carriers and polyphenols, in our case apple fibers and blackberry juice, it can be expected that adsorption of volatiles of blackberry juice onto apple fiber can occur. For this reason, we conducted an analysis of volatile compounds in blackberry juice, apple fiber and apple fiber/blackberry juice complexes.

**Figure 1:**
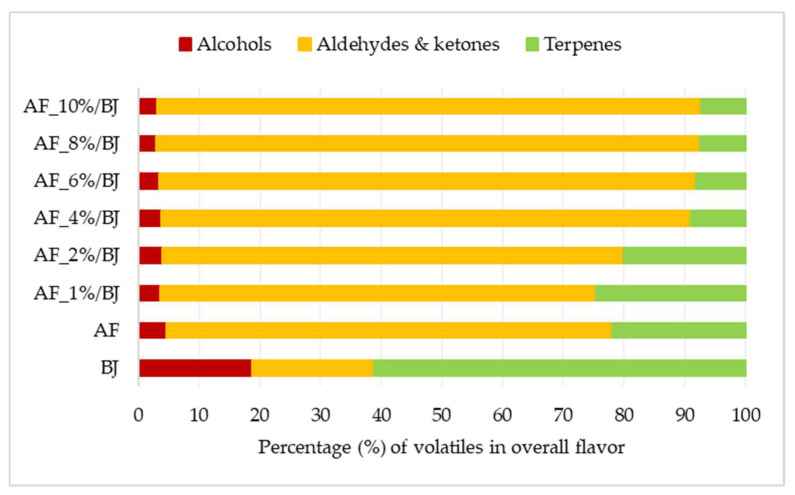
Percentage of different chemical classes of volatiles in overall flavor amount of blackberry juice, apple fiber and apple fiber/blackberry juice complexes (AF—apple fiber; BJ—blackberry juice; 1–10%—amounts of AF).

**Figure 2:**
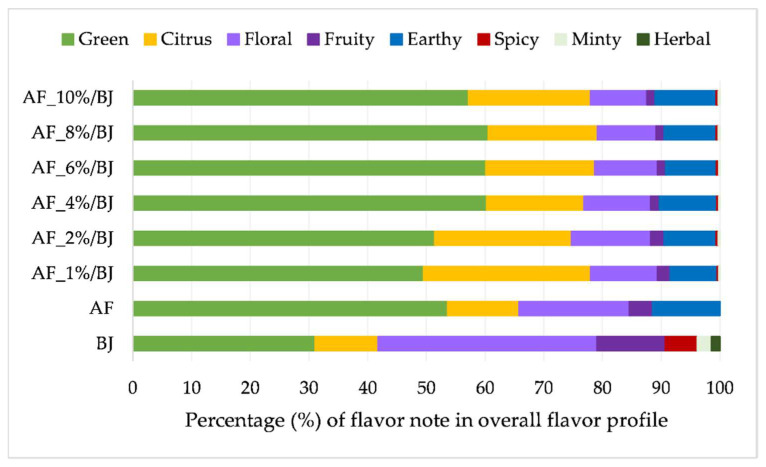
Percentage of specific flavor note in the overall flavor profile of blackberry juice, apple fiber and apple fiber/blackberry juice complexes (AF—apple fiber; BJ—blackberry juice; 1–10%—amounts of AF).

Volatiles are divided into three chemical classes, that is, alcohols, terpenes and aldehydes, and ketones and their contribution to the overall flavor amount are presented in Figure 1 (amounts of individual volatiles are presented in Supplementary Materials). For blackberry juice, it can be observed that terpenes are contributing the most to the flavor amount at 61%, while the contribution of the other two chemical classes was equal (around 20%). Aldehydes and ketones contributed mostly to the apple flavor amount (74%) while the contribution of terpenes was 22% and only 5% for alcohols. The flavor of apple fiber/blackberry juice complexes consisted of the flavor of both ingredients used for complexation but some of them were lost during preparation. The ratio between determined volatiles changed in the obtained complexes. The contribution of alcohols to the overall flavor amount for all complexes was equal, 4%. Complexes prepared with 4% to 10% of apple fiber had terpenes from 7% to 9% and aldehydes and ketones from 87% to 90%. The other two complexes had a higher amount of terpenes 25% and 20%, and a lower amount of aldehydes and ketones 72% and 76%, than before mentioned complexes. Additionally, the flavor profile was examined on our samples. Each volatile compound is characterized by its flavor note thus volatiles were grouped accordingly into green, citrus, floral, fruity, earthy, spicy, minty and herbal flavor notes. The contribution of each flavor note to the overall flavor profile of blackberry juice, apple fiber and apple fiber/blackberry juice complexes is presented in Figure 2. The most dominant flavor note for blackberry juice was floral (37%), followed by green (31%) and citrus and fruity (each contributing with 11%). Apple fiber had a different flavor profile with the dominant green flavor note (53%), followed by floral (18%) and citrus and earthy (each contributing with 11%). Consequently, the flavor profile of apple fiber/blackberry juice complexes was a combination of both ingredients used for its preparation. The most dominant flavor note on complexes was green flavor note (from 50% to 60%), followed by citrus (from 16% to 28%), floral (from 10% to 13%) and earthy (from 8% to 10%) flavor note. Other studies also proved that during the complexation of raspberry juice with cellulose [47], adsorption of volatile compounds occurred influencing also flavor profile of complexes not only on their color and phenolic profile.

### 2.5. FTIR-ATR Analysis of Apple Fiber/Blackberry Juice Complexes

**Figure 3:**
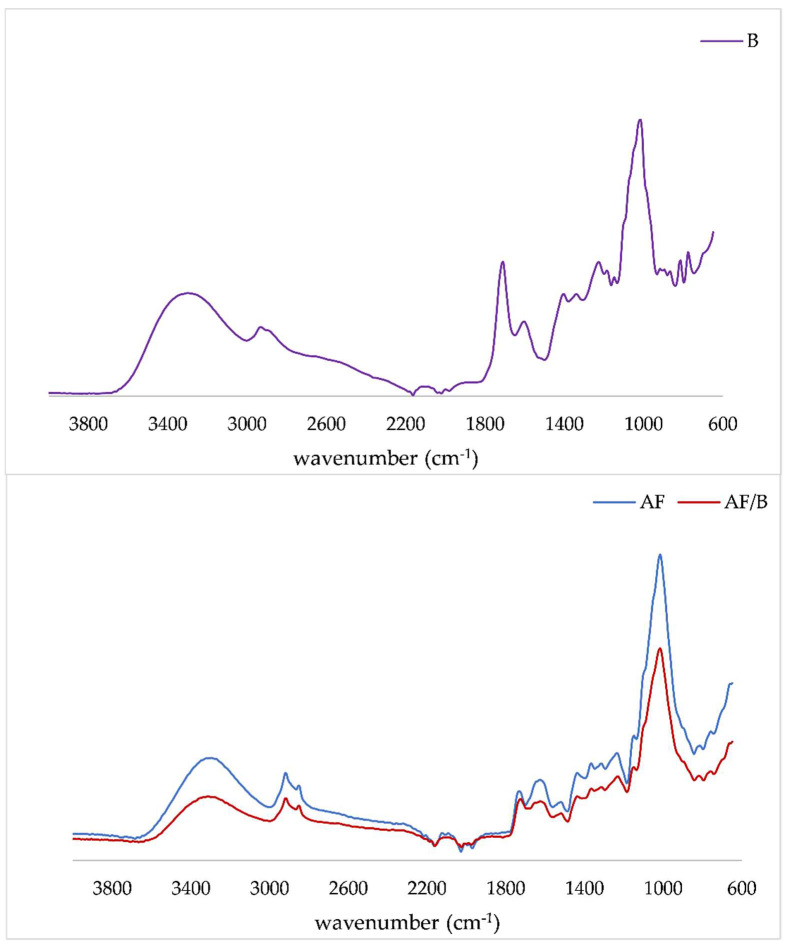
IR spectra of blackberry juice (B), apple fiber (AF) and apple fiber/blackberry juice complex (AF/B).

FTIR-ATR technique was used to detect changes in the main components of apple fibers as well their modification caused by the adsorption of polyphenols. IR spectra of apple fiber, as well as of the obtained complexes, are presented in Figure 3. Only one IR spectrum of the complex was presented because there were no differences between complexes.

The region between 3200 cm^−1^ and 3600 cm^−1^ could be assigned to the intermolecular bound and free hydroxyl groups, associated with the O-H groups of fibers. The bands at 2922 cm^−1^ and 2850 cm^−1^ are assigned to the C–H stretching vibration [48]. Furthermore, the band at 1730 cm^−1^ is connected to the C = O stretching vibration of alkyl ester of pectin, while the band at 1625 cm^−1^ to COO- antisymmetric stretching [49]. The bands at 1438 cm^−1^ and 1308 cm^−1^ can be associated with CH_2_ symmetric bending in cellulose, while the band at 1372 cm^−1^ is assigned to out-of-plane bending vibrations of C–H bonds of the cellulose glycoside ring. The band at 1013 cm^−1^ can be assigned to C–O stretching and C–C stretching at pectin. All bands in the range from 600 cm^−1^ to 900 cm^−1^ are connected to C–H out-of-plane bending vibrations [48,49,50]. The main observation was that the band intensities in the IR spectrum decreased when polyphenols from blackberry juice were loaded. This finding was in accordance with studies by Abdelwahab and Amin [51]. These authors investigated the interactions between Luffa cylindrical fibers and polyphenols and observed a decrease in band intensities when polyphenols penetrated the interlayer space of fiber. A similar observation was in our previously published paper [26] where a decrease in band intensity was noticed when polyphenols were encapsulated onto citrus fibers, especially the intensity of the band at around 1610 cm^−1^. Also, in the present study, on apple fiber, the intensity of the band at 1625 cm^−1^ was higher, compared to 1730 cm^−1^, while on complexes due to adsorption of polyphenols, a reverse trend was observed. Furthermore, Vukoja et al. [20] observed a decrease in hydrogen-bond intensity in the IR spectrum of encapsulates (raspberry polyphenols with cellulose) compared to pure cellulose.

## 3. Materials and Methods

### 3.1. Materials

Apple fibers were obtained from Biesterfeld AG (Zagreb, Croatia). 2,2′-azino-bis(3-ethylbenzothiazoline-6-sulfonic acid) diammonium salt (ABTS), 4-(dimethylamino)-cinnamaldehyde (DMAC), trolox, 2,2-diphenyl-1-picrylhydrazyl (DPPH), rutin, quercetin, phloretin, phlorizin, chlorogenic, caffeic, p-coumaric, gallic, ellagic acids, myrtenol and α-amylase (from the porcine pancreas) were obtained from Sigma-Aldrich (St. Louis, MO, USA). Potassium persulfate, Folin–Ciocalteu reagent and sodium carbonate were purchased from Kemika (Zagreb, Croatia). Neocuproine, 2,4,6-tri(2-pyridyl)-s-triazine (TPTZ) and copper (II) chloride were bought from Acros Organics (Geel, Belgium). Methanol (HPLC grade) was from J.T. Baker (Deventer, Netherlands) and orthophosphoric acid (HPLC grade > 85%) was from Fisher Scientific (Loughborough, UK). Iron (III) chloride hexahydrate, sodium acetate, ethanol, ammonium acetate and starch were bought from Gram-mol (Zagreb, Croatia). Cyanidin 3-glucoside was from Extrasynthese (Genay, France) and 3,5-dinitrosalicylic acid from Alfa Aesar (Kandel, Germany).

### 3.2. Preparation of Apple Fiber/Blackberry Juice Complexes

Ripe blackberry fruits were pressed to obtain juice, which then was filtered through cheesecloth to remove solids. The obtained juice was thermally treated at 90 °C for 3 min to inactivate the enzymes. Apple fibers were added (1%, 2%, 4%, 6%, 8% or 10%) to blackberry juice (50 mL) and mixed using a magnetic stirrer (Stuart US152, Buch and Holm, Hervel, Denmark) for 15 min at room temperature. After mixing, the mixtures were centrifuged (15 min; 2540× *g*) (Microspin 12, Grant Instruments Ltd., Royston, UK). The supernatant was discarded and the precipitate was frozen at −18 °C. After 24 h, the precipitate was lyophilized in the laboratory freeze-dryer (Christ Freeze Dryer, Alpha 1–4, Osterode am Harz, Germany) for 12 h. The temperature of freezing was −55 °C, while the temperature of sublimation was from −35 °C to 0 °C and the vacuum level was 0.220 mbar. The isothermal desorption temperatures ranged from 0 °C to 21 °C under vacuum (0.060 mbar). The obtained complexes were further analyzed.

### 3.3. Extraction of Complexes

Approximately 0.2 g of the formed complex was extracted with 10 mL of acidified methanol (methanol:HCl ratio was 99:1 (*v*/*v*)). Extraction was conducted for 24 h at room temperature, after which the mixture was filtered. The extracts were used for spectrophotometric determination of total polyphenols content, proanthocyanidins content, antioxidant activities and inhibition of α-amylase as well as HPLC determination of individual polyphenols.

### 3.4. Evaluation of Total Polyphenols and Proanthocyanidins Contents

A colorimetric Folin–Ciocalteu method [52] was used for the evaluation of total polyphenols. Briefly, 0.2 mL of the extract was added in a glass tube together with 1.8 mL of deionized water, afterward 10 mL (1:10) of Folin–Ciocalteu reagent and 8 mL of sodium carbonate (7.5%) were added. The mixture was incubated at room temperature for 120 min and then the absorbance was measured at 765 nm on a UV/Vis spectrophotometer (Cary 60 UV-Vis, Agilent Technologies, Santa Clara, CA, USA) and the results were expressed as g gallic acid equivalents per 100 g of sample (g GAE/100 g).

DMAC method [53] was used for the evaluation of proanthocyanidins content. An aliquot of the extract was mixed with 4-(dimethylamino)-cinnamaldehyde solution and after 30 min the absorbance was measured at 640 nm. The results were expressed as mg of procyanidin B2 equivalent per 100 g of sample (mg PB2E/100 g). Measurements were conducted in triplicate.

### 3.5. High-Performance Liquid Chromatography (HPLC) Analysis

Agilent HPLC system 1260 Infinity II (Agilent Technology, Santa Clara, CA, USA) equipped with a quaternary pump, a diode array detector (DAD), a vial sampler and a Poroshell 120 EC C-18 column (4.6 × 100 mm, 2.7 µm) was used for determination of anthocyanins, phenolic acids, flavanols and dihydrochalcones in complexes. Prior to injection, 1 mL of extract was filtered through a 0.20 µm PTFE syringe filter. Orthophosphoric acid (0.1%) was used as the mobile phase A and HPLC grade methanol (100%) as the mobile phase B. For separation, the following gradient was used: 0 min 5% B, 3 min 30% B, 15 min 35% B, 22 min 37% B, 30 min 41% B, 32 min 45% B, 40 min 49% B, 45 min 80% B, 48 min 80% B, 50 min 5% B, 53 min 5% B. The stock solutions of the polyphenol standards were used for calibration curves construction at the following concentrations range and linearity: cyanidin 3-glucoside (10–500 mg/L; r^2^ = 0.9992), ellagic acid (5–50 mg/L; r^2^ = 0.9989), chlorogenic acid (25–500 mg/L; r^2^ = 0.999), caffeic acid (0.25–300 mg/L; r^2^ = 0.9997), gallic acid (25–300 mg/L; r^2^ = 0.9986), p-coumaric acid (25–300 mg/L; r^2^ = 0.9992), rutin (0.25–500 mg/L; r^2^ = 0.9993) quercetin (5–150 mg/L; r^2^ = 0.9998), phloretin (0.5–50 mg/L; r^2^ = 0.9997) and phlorizin (1–50 mg/L; r^2^ = 0.9998). In the range of 190 to 600 nm, UV/Vis spectra were recorded and used for identification of peaks in the extract. Also, retention times of standards were compared with those in extracts and extracts were spiked with standards to confirm identification. Cyanidin 3-dioxalylglucoside and two derivates of hydroxycinnamic acid were tentatively identified according to the literature data [27] and for quantification, calibration curves of cyanidin 3-glucoside and chlorogenic acid were used.

### 3.6. Determination of Antioxidant Activity

For the evaluation of the antioxidant activity of complexes, four spectrophotometric assays (ABTS, DPPH, CUPRAC and FRAP) were used. The ABTS assay was performed according to Arnao et al. [54] with modifications. The extract (0.2 mL) was mixed with 3.2 mL of ABTS reagent and after 95 min, absorbance was recorded at 734 nm. The DPPH assay was conducted according to Brand-Williams et al. [55] with slight modifications. Briefly, 0.2 mL of extract was added to a glass tube, followed by the addition of 3 mL 0.5 mM DPPH solution. This mixture remained for 15 min in the dark and then its absorbance was recorded at 517 nm. CUPRAC (cupric reducing antioxidant capacity) assay was performed according to Apak et al. [56]. To a glass tube, 1 mL of CuCl_2_ (10 mM), neocuproine (7.5 mM) and ammonium acetate buffer (1 M, pH 7.0) were added, followed by the addition of the extract and distilled water in a total volume of 1.1 mL. After 30 min in the dark, the absorbance was read at 450 nm. Ferric reducing ability power (FRAP) was examined using the protocol described by Benzie and Strain [57]. The mixture of 0.2 mL of extract and 3 mL of FRAP reagent was measured at 593 nm after 30 min of incubation. Antioxidant activity assays were performed in triplicate and the results were expressed as micromoles of Trolox equivalent per 100 g of sample (µmol TE/100 g) (for ABTS, DPPH, CUPRAC and FRAP assays).

### 3.7. Inhibition of α-Amylase

Inhibition of α-amylase measurements was performed according to the protocol described by Da Silva et al. [58] and Kellogg et al. [59] with slight modifications. In short, 0.2 mL of extract was added to a glass tube followed by the addition of 0.4 mL of α-amylase solution (1 mg/mL). After 10 min of incubation at 37 °C, 0.2 mL of starch solution (1%) was added and the mixture was re-incubated. The reaction was stopped with the addition of 1 mL of 3,5-dinitrosallicylic acid (DNS) reagent and boiling for 5 min. Then, the mixture was cooled at room temperature, 10 mL of distilled water was added and absorbance was measured at 540 nm. A control sample (uninhibited reaction) and a blank (without the enzyme present) were also measured for each sample. The percentage of inhibition was calculated according to the following formula:

I(%) = (A_inh_ − A_blank_)/A_con_ × 100 (1)

where A_inh_ was the absorbance of the inhibited reaction, A_blank_ absorbance of the extract with substrate (no enzyme present) and A_con_ absorbance of the uninhibited enzyme.

### 3.8. Color Evaluation

Color parameters of apple fiber/blackberry juice complexes were measured by Minolta Chroma meter CR-400 (Minolta; Osaka, Japan) using the Lab system. The explanation of the determined color parameters is as follows: L*—lightness (0—black and 100—white); a*—(+) redness and (−) greenness; b*—(+) yellowness and (−) blueness; C*—value of the color saturation (chroma) and °h—the hue angle (from 0° for red, over 90° for yellow and 180° for green, up to 270° for blue and back to 0°).

### 3.9. Evaluation of Volatile Compounds

Volatiles from formulated complexes were extracted by solid-phase microextraction (SPME). 0.3 g of sample, 4.7 g of water and 1 g of NaCl were weighed into a 10 mL glass vial. The details of the method have been explained in a previous study by Vukoja et al. [47].

### 3.10. Fourier Transform Infrared with Attenuated Total Reflection (FTIR-ATR) Spectroscopy Analysis

The FTIR-ATR (Cary 630, Agilent, Santa Clara, CA, USA) equipped with the software MicroLab Expert was used to obtain the infrared spectra of complexes. IR spectra were recorded in the range from 4000 cm^−1^ to 600 cm^−1^.

### 3.11. Statistical Analysis

Statistical analysis was performed using the software program STATISTICA 13.1 (StatSoft Inc., Tulsa, OK, USA), by analysis of variance (ANOVA) and Fisher’s least significant difference (LSD) test, with the significance defined at *p* < 0.05.

## 4. Conclusions

Apple fibers could be used for the encapsulation of blackberry juice polyphenols but only under certain conditions. These conditions include the amount of used fiber as the main factor for maximizing the retention of polyphenols. Complex with 1% of fiber possessed the highest total polyphenols content, proanthocyanidins content and antioxidant activities. However, the sum of individual polyphenols determined by the HPLC analysis showed the highest concentration in the complex with 2% of fiber. Our results showed that both the individual polyphenols from blackberry juice and apple fiber as well as their ratios affected the antioxidant activity and inhibition of α-amylase. Also, it was proved that volatile compounds from blackberry juice adsorbed onto apple fiber affected the flavor profile of the complexes. Future studies will be focused on the application of these additives in the actual food matrices as colorants, antioxidants and flavoring agents.

## Figures and Tables

**Table 1:** Total polyphenols content (TPC) and total proanthocyanidins content (PAC) in apple fiber/blackberry juice complexes.

	TPC (g GAE/100 g)	PAC (mg PB2E/100 g)
AF_1%/BJ	1.82 ± 0.00 ^a^	98.85 ± 1.35 ^a^
AF_2%/BJ	1.79 ± 0.01 ^b^	93.51 ± 2.93 ^a^
AF_4%/BJ	1.50 ± 0.01 ^c^	88.56 ± 0.47 ^b^
AF_6%/BJ	1.28 ± 0.02 ^d^	85.39 ± 2.68 ^b^
AF_8%/BJ	1.25 ± 0.01 ^e^	73.47 ± 1.96 ^c^
AF_10%/BJ	1.18 ± 0.01 ^f^	57.43 ± 2.71 ^d^

AF—apple fiber; BJ—blackberry juice; 1–10%—amounts of AF; GAE—gallic acid equivalents; PB2E—procyanidin B2 equivalents. Values (^a–f^) in the same column marked with different superscripts are statistically different at *p* ≤ 0.05 (ANOVA, Fisher’s LSD).

**Table 2:** Characterization of blackberry juice and apple fiber.

Blackberry Juice		Apple Fiber	
Individual Polyphenols (mg/100 g)			
C 3-G	339.8 ± 0.40	Que	130.60 ± 5.38
C 3-DG	118.8 ± 0.03	Rut	9.54 ± 1.14
Que	22.7 ± 0.05	ChA	51.64 ± 2.70
Rut	3.7 ± 0.00	HC-1	17.36 ± 0.13
EA	27.35 ± 0.00	HC-2	15.72 ± 0.02
CA	3.8 ± 0.00	P	17.64 ± 0.48
ChA	31.55 ± 0.03	Ph	78.21 ± 0.60
p-CA	41.1 ± 0.00		
GA	36.3 ± 0.01		
TPC (mg GAE/100 g)	58.33 ± 0.05		1136.76 ± 15.50
PAC (mg PB2E/100 g)	2.07 ± 0.05		153.69 ± 1.65
ABTS (µmol TE/100 g)	5.82 ± 0.02		50.37 ± 0.46
DPPH (µmol TE/100 g)	2.33 ± 0.01		54.30 ± 0.34
CUPRAC (µmol TE/100 g)	24.04 ± 0.68		532.80 ± 15.99
FRAP (µmol TE/100 g)	0.47 ± 0.00		6.94 ± 0.11

C 3-G—cyanidin 3-glucoside; C 3-DG—cyanidin 3-dioxalylglucoside; EA—ellagic acid; CA—caffeic acid, ChA—chlorogenic acid; p-CA—p-coumaric acid; GA—gallic acid; Que—quercetin; Rut—rutin; P—phloretin; Ph—phlorizin; HC-1 and HC-2—two derivates of hydroxycinnamic acid; TPC—total polyphenols content; (PAC—proanthocyanidins content; DPPH (2,2-diphenyl-1-picrylhydrazyl)—free radical scavenging activity; ABTS (2,20-azino-bis(3-ethylbenzothiazoline-6-sulfonic acid)—radical scavenging activity; FRAP—ferric reducing antioxidant power; CUPRAC—cupric reducing antioxidant capacity; TE—trolox equivalents; PB2E—procyanidin B2 equivalents.

**Table 3:** Individual polyphenols content (mg/100 g) in apple fiber/blackberry juice complexes.

	AF_1%/BJ	AF_2%/BJ	AF_4%/BJ	AF_6%/BJ	AF_8%/BJ	AF_10%/BJ
*Anthocyanins*						
C 3-G	208.01 ± 1.56 ^b^	219.63 ± 2.85 ^a^	152.88 ± 2.24 ^c^	131.19 ± 0.51 ^d^	109.49 ± 1.32 ^e^	103.72 ± 0.05 ^f^
C 3-DG	54.35 ± 0.38 ^b^	57.81 ± 0.93 ^a^	43.58 ± 0.70 ^c^	39.02 ± 0.04 ^d^	33.60 ± 0.04 ^e^	31.94 ± 0.04 ^f^
*Total*	*262.36 ± 1.94 ^b^*	*277.44 ± 3.78 ^a^*	*196.46 ± 2.94 ^c^*	*170.21 ± 0.55 ^d^*	*143.09 ± 1.36 ^e^*	*135.66 ± 0.09 ^e^*
*Flavanols*						
Que	55.60 ± 0.41 ^f^	70.39 ± 0.88 ^e^	80.73 ± 0.67 ^d^	89.87 ± 0.87 ^c^	94.14 ± 0.26 ^b^	96.82 ± 0.55 ^a^
*Phenolic acids*						
EA	37.97 ± 0.38 ^a^	35.82 ± 0.29 ^b^	23.71 ± 0.17 ^c^	20.51 ± 0.03 ^d^	15.53 ± 0.32 ^e^	15.25 ± 0.02 ^e^
ChA	17.42 ± 0.05 ^f^	19.60 ± 0.02 ^e^	21.84 ± 0.18 ^d^	23.99 ± 0.01 ^c^	24.65 ±0.28 ^b^	26.07 ± 0.08 ^a^
HC-1	18.00 ± 0.16 ^c,d^	18.14 ± 0.16 ^b,d^	18.26 ± 0.01 ^b^	18.73 ± 0.10 ^a^	18.25 ± 0.08 ^b,c^	18.41 ± 0.02 ^b^
HC-2	16.46 ± 0.04 ^a,b^	16.22 ± 0.33 ^b,c^	16.34 ± 0.04 ^a,c^	16.60 ± 0.02 ^a^	16.10 ± 0.03 ^c^	16.10 ± 0.00 ^c^
*Total*	*89.85 ± 0.63 ^a^*	*89.78 ± 0.80 ^a^*	*80.15 ± 0.40 ^b^*	*79.83 ± 0.16 ^b^*	*74.53 ± 0.71 ^c^*	*75.83 ± 0.12 ^c^*
*Dihydrochalcones*						
P	4.06 ± 0.06 ^f^	5.49 ± 0.02 ^e^	7.57 ± 0.22 ^d^	9.03 ± 0.26 ^c^	9.86 ± 0.32 ^b^	11.26 ± 0.39 ^a^
Ph	15.23 ± 0.03 ^f^	22.47 ± 0.18 ^e^	30.99 ± 0.64 ^a^	36.27 ± 0.11 ^c^	38.80 ± 0.42 ^b^	41.86 ± 0.30 ^a^
*Total*	*19.29 ± 0.09 ^f^*	*27.96 ± 0.20 ^e^*	*38.56 ± 0.86 ^d^*	*45.30 ± 0.37 ^c^*	*48.66 ± 0.74 ^b^*	*53.12 ± 0.69 ^a^*
*TOTAL*	*427.10 ± 3.07 ^b^*	*465.57 ± 5.66 ^a^*	*395.90 ± 4.87 ^c^*	*385.21 ± 1.95 ^c^*	*360.42 ± 3.07 ^d^*	*361.43 ± 1.45 ^d^*

C 3-G—cyanidin 3-glucoside; C 3-DG—cyanidin 3-dioxalylglucoside; Que—quercetin; EA—ellagic acid; ChA—chlorogenic acid; HC-1 and HC-2—two derivates of hydroxycinnamic acid; P—phloretin; Ph—phlorizin; AF—apple fiber; BJ—blackberry juice; 1–10%—amounts of AF. Values in the same row marked with different superscripts are statistically different at *p* ≤ 0.05 (ANOVA, Fisher’s LSD).

**Table 4:** Antioxidant activity of apple fiber/blackberry juice complexes and inhibition of α-amylase enzyme.

	ABTS (µmol TE/100 g)	DPPH (µmol TE/100 g)	CUPRAC (µmol TE/100 g)	FRAP (µmol TE/100 g)	α-Amylase Inhibition (%)
AF_1%/BJ	106.86 ± 0.12 ^a^	109.64 ± 0.12 ^a^	964.00 ± 3.08 ^a^	13.19 ± 0.11 ^a^	33.05 ± 0.67 ^c^
AF_2%/BJ	95.85 ± 0.79 ^b^	101.01 ± 0.17 ^b^	888.65 ± 3.76 ^b^	11.81 ± 0.14 ^b^	35.90 ± 0.37 ^b^
AF_4%/BJ	82.77 ± 0.42 ^c^	100.45 ± 0.64 ^b,c^	848.27 ± 2.93 ^c^	11.46 ± 0.10 ^b^	37.53 ± 0.10 ^a^
AF_6%/BJ	82.27 ± 0.47 ^c^	98.81 ± 0.56 ^c^	830.95 ± 2.68 ^d^	10.70 ± 0.18 ^c^	33.14 ± 0.23 ^c^
AF_8%/BJ	77.62 ± 0.31 ^d^	95.58 ± 0.83 ^d^	790.74 ± 2.52 ^e^	10.25 ± 0.12 ^c^	33.56 ± 0.29 ^c^
AF_10%/BJ	73.74 ± 0.86 ^e^	88.90 ± 0.45 ^e^	687.97 ± 2.15 ^f^	9.04 ± 0.05 ^d^	22.10 ± 0.21 ^d^

AF—apple fiber; BJ—blackberry juice; 1–10%—amounts of AF; DPPH (2,2-diphenyl-1-picrylhydrazyl)—free radical scavenging activity; ABTS (2,20-azino-bis(3-ethylbenzothiazoline-6-sulfonic acid)—radical scavenging activity; FRAP—ferric reducing antioxidant power; CUPRAC—cupric reducing antioxidant capacity; TE—trolox equivalents. Values in the same column marked with different superscripts are statistically different at *p* ≤ 0.05 (ANOVA, Fisher’s LSD).

**Table 5:** Color parameters of apple fiber/blackberry juice complexes.

	L*	a*	b*	°h	C*
AF_1%/BJ	45.90 ± 0.09 ^f^	18.03 ± 0.08 ^d^	8.77 ± 0.01 ^f^	25.92 ± 0.07 ^f^	20.01 ± 0.11 ^e^
AF_2%/BJ	46.71 ± 0.03 ^e^	19.15 ± 0.04 ^b^	9.76 ± 0.01 ^e^	26.99 ± 0.08 ^e^	21.49 ± 0.03 ^d^
AF_4%/BJ	47.89 ± 0.02 ^d^	19.41 ± 0.01 ^a^	10.93 ± 0.02 ^d^	29.38 ± 0.03 ^d^	22.27 ± 0.02 ^c^
AF_6%/BJ	48.41 ± 0.01 ^c^	19.33 ± 0.03 ^a^	11.90 ± 0.01 ^c^	31.60 ± 0.03 ^c^	22.70 ± 0.03 ^a,b^
AF_8%/BJ	49.30 ± 0.02 ^b^	18.99 ± 0.03 ^b^	12.76 ± 0.03 ^b^	33.89 ± 0.10 ^b^	22.88 ± 0.02 ^a^
AF_10%/BJ	49.70 ± 0.01 ^a^	18.48 ± 0.06 ^c^	12.93 ± 0.01 ^a^	34.98 ± 0.08 ^a^	22.54 ± 0.06 ^b^

AF—apple fiber; BJ—blackberry juice; 1–10%—amounts of AF. Values in the same column marked with different superscripts are statistically different at *p* ≤ 0.05 (ANOVA, Fisher’s LSD).

## Data Availability

The data presented in this study are available in Supplementary Material.

## Supplementary Materials

The following supporting information can be downloaded at: https://www.mdpi.com/article/10.3390/molecules27093029/s1, Table S1: Volatile compounds identified in blackberry juice (BJ), apple fibers (AF) and apple fiber/blackberry juice complexes (AF/BJ) with their odor descriptor; Table S2: Amount of volatile compounds on apple fiber/blackberry juice complexes.

## Sample Availability

Samples of the compounds of apple fiber are available from the authors.
