# Supplementary material for: Apple Fibers as Carriers of Blackberry Juice Polyphenols: Development of Natural Functional Food Additives
Source: Molecules. 2022 May 8;27(9):3029. doi: 10.3390/molecules27093029 (PMC9101031; doi:10.3390/molecules27093029)
Supplement: Supplementary file 1 [file molecules-27-03029-s001.zip › molecules-1712973-supplementary.pdf]

Table S1. Volatile compounds identified in blackberry juice (BJ), apple fibers (AF) and apple fiber/blackberry juice complexes (AF/BJ) with their odor descriptor

Volatiles	BJ	AF	AF/BJ	Odor descriptor
Alcohols				
1-butanol	+	-	+	fruity
2-ethylhexanol	+	+	+	fruity
Benzyl alcohol	-	-	+	fruity
1-octanol	+	+	+	green
Phenethyl alcohol	+	-	+	floral
Perillyl alcohol	+	+	+	green
Aldehydes and ketones				
Hexanal	-	+	+	green
Heptanal	+	+	+	green
2-heptenal	-	+	+	green
1-octen-3-one	-	+	+	earthy
6-methyl-5-hepten-2-on	-	-	+	green
2,4-heptadienal	-	-	+	citrus
Octanal	-	-	+	green
2-octenal	+	+	+	green
2,6-nonadienal	-	-	+	floral
Nonanal	+	+	+	floral
2-nonenal	-	+	+	green
Decanal	-	+	+	floral
2,4-nonadienal	+	+	+	green
2-decenal	+	+	+	citrus
4-propylbenzaldehyde	+	-	+	-
2,4-decadienal	+	+	+	citrus
Geranylacetone	+	+	+	floral
Lily aldehyde	+	-	-	floral
α-hexylcinnamal	+	-	-	floral
Terpenes				
D-limonene	+	+	+	citrus
Citronellal	+	+	+	floral
Guaiacol	+	-	-	floral
Linalool	+	-	+	green
Trans-verbenol	+	+	+	floral
Menthol	+	-	+	-
Nerol	+	-	+	minty
Citral	+	-	+	citrus
D-limonene	+	-	-	herbal
Citronellal	+	+	+	citrus
Vitispirane	+	-	-	floral
Eugenol	+	-	+	spicy
β-damascenon	+	+	+	fruity
α-ionon	+	+	+	fruity
γ-ionone	+	+	+	fruity
β-ionone	+	+	+	fruity

Table S2. Amount of volatile compounds on apple fiber/blackberry juice complexes

Volatiles	Complexes					
	AF_1%/BJ	AF_2%/BJ	AF_4%/BJ	AF_6%/BJ	AF_8%/BJ	AF_10%/BJ
Alcohols						
2-ethylhexanol	6.71±0.38^{c}	8.34±0.12^{a}	8.63±0.14^{a}	6.74±0.09^{c}	6.00±0.15^{d}	7.23±0.31^{b}
Benzyl alcohol	26.26±1.02^{a}	24.59±0.15^{a}	18.54±0.12^{b}	16.57±0.57^{c}	15.35±0.11^{d}	15.98±0.18$^{c.d}$
1-octanol	44.42±0.56^{e}	52.41±1.90^{d}	93.35±2.18^{a}	94.08±1.27^{a}	74.09±0.23^{c}	86.56±0.45^{b}
Phenethyl alcohol	17.35±0.19^{e}	22.45±0.33^{d}	23.67±0.35^{c}	26.92±0.19^{a}	26.06±0.08^{b}	27.55±0.19^{a}
Perillyl alcohol	6.55±0.25^{e}	8.24±0.34^{d}	11.31±0.42^{c}	13.39±0.17^{b}	13.20±0.24^{b}	14.91±0.24^{a}
Aldehydes and ketones						
Hexanal	108.66±4.10^{e}	104.42±0.48^{e}	139.25±2.35^{d}	150.07±1.26^{c}	158.90±2.33^{b}	297.81±4.57^{a}
Heptanal	1.24±0.07^{e}	5.50±0.05^{c}	11.50±0.28^{a}	9.45±0.43^{b}	4.96±0.16^{d}	4.74±0.28^{d}
2-heptenal	791.25±13.38^{c}	734.65±8.01^{d}	1252.53±7.37^{b}	1412.70±46.47^{a}	1452.75±1.99^{a}	1456.35±3.01^{a}
1-octen-3-one	210.66±1.53^{e}	237.30±1.83^{d}	366.10±29.68^{c}	366.49±7.93^{c}	373.98±3.26^{b}	470.55±7.00^{a}
6-methyl-5-hepten-2-on	71.29±2.77^{c}	88.86±1.17^{a}	89.00±0.41^{a}	80.33±0.82^{b}	81.63±4.28^{b}	82.01±0.93^{b}
2,4-heptadienal	38.00±1.15^{c}	39.11±0.22^{c}	56.26±2.12^{a}	49.03±0.54^{b}	47.40±0.83^{b}	47.79±3.17^{b}
Octanal	79.67±2.37^{e}	126.32±2.85^{d}	135.92±2.58^{c}	141.44±0.74^{b}	142.31±1.23$^{a.b}$	145.75±2.73^{a}
2-octenal	301.62±11.37^{d}	391.79±21.97^{c}	634.65±46.57^{b}	748.43±3.67^{a}	725.79±16.84^{a}	732.36±8.80^{a}
2,6-nonadienal	5.79±0.14^{d}	7.26±0.14^{c}	7.29±0.06^{c}	8.42±0.18^{b}	8.47±0.11^{b}	9.05±0.16^{a}
Nonanal	92.35±0.51^{e}	113.00±1.07^{d}	167.88±1.45^{c}	199.50±3.14^{a}	171.56±0.28^{b}	174.04±1.60^{b}
2-nonenal	46.60±0.87^{f}	55.20±3.58^{e}	86.46±1.90^{d}	105.60±0.19^{b}	101.10±0.12^{c}	110.32±0.62^{a}
Decanal	35.79±0.13^{d}	44.15±1.02^{c}	50.53±1.67^{b}	55.79±1.02^{a}	56.44±0.55^{a}	56.24±1.35^{a}
2,4-nonadienal	25.29±0.50^{d}	28.65±1.39^{c}	79.11±2.06^{b}	79.78±0.70^{b}	80.48±0.91^{b}	86.35±0.19^{a}
2-decenal	180.51±1.73^{e}	219.85±1.60^{d}	437.48±6.61^{c}	608.70±14.60^{b}	629.12±18.32^{b}	772.34±53.57^{a}
4-propylbenzaldehyde	21.19±0.16^{a}	18.08±0.04^{b}	17.30±0.23^{b}	17.48±0.25^{b}	17.64±0.05^{b}	17.91±0.41^{b}
2,4-decadienal	17.49±0.44^{e}	28.16±1.49^{d}	40.45±1.07^{c}	50.03±1.06^{b}	50.16±1.64^{b}	54.42±0.69^{a}
Geranylacetone	46.72±0.70^{a}	37.24±2.18^{b}	39.97±1.97^{b}	26.03±0.31^{c}	23.37±0.03^{d}	23.90±0.91^{d}
Terpenes						
D-limonene	549.94±40.04^{a}	363.70±27.55^{b}	130.46±1.65^{c}	105.86±6.34^{d}	80.18±4.75^{e}	85.96±0.97^{e}
Citronellal	49.87±1.30^{a}	51.20±0.49^{a}	47.85±0.03^{b}	41.38±0.84^{c}	41.71±1.03^{c}	47.83±1.03^{b}
Guaiacol	18.26±0.10^{c}	19.97±0.03^{b}	19.33±0.45^{b}	22.06±0.04^{a}	21.88±0.08^{a}	21.66±0.40^{a}
Linalool	52.66±0.65^{d}	88.73±1.32^{c}	89.22±0.25^{c}	96.11±1.35^{a}	92.99±0.18^{b}	97.46±1.25^{a}
Trans-verbenol	2.43±0.04^{c}	2.97±0.03^{c}	3.39±0.34^{b}	4.22±0.08^{a}	4.42±0.06^{a}	3.66±0.02^{b}
Menthol	4.63±0.11^{e}	6.89±0.61^{d}	7.75±0.15^{c}	9.01±0.25^{b}	9.79±0.20^{b}	11.51±0.07^{a}
Nerol	-	3.38±0.11^{e}	7.70±0.06^{d}	13.18±0.01^{b}	12.52±0.17^{c}	15.46±0.07^{a}
Citral	3.90±0.11^{c}	8.79±0.15^{b}	8.41±0.15^{b}	10.01±0.05^{a}	9.87±0.59^{a}	10.91±0.66^{a}
Eugenol	7.98±0.14^{e}	10.14±0.27^{d}	12.27±0.14^{c}	15.82±0.02^{b}	15.86±0.44^{b}	17.48±0.68^{a}
β-damascenon	5.90±0.00^{d}	9.13±0.05^{c}	9.09±0.01^{c}	9.81±0.01^{b}	9.76±0.34^{b}	12.82±0.20^{a}
α-ionon	2.38±0.04^{d}	2.75±0.00^{c}	2.90±0.12^{c}	3.85±0.04^{b}	3.54±0.24^{b}	4.22±0.06^{a}
γ-ionone	6.21±0.04^{c}	5.73±0.10^{d}	8.17±0.35^{b}	10.85±0.09^{a}	10.86±0.55^{a}	10.39±0.03^{a}
β-ionone	9.10±0.13^{e}	10.08±0.06^{d}	10.25±0.01^{d}	12.12±0.10^{b}	11.75±0.11^{c}	12.81±0.19^{a}

Values in the same raw marked with different superscripts are statistically different at $p \leq 0.05$ (ANOVA, Fisher's LSD).
